# *De Novo* Assembly of the Transcriptome of the Non-Model Plant *Streptocarpus rexii* Employing a Novel Heuristic to Recover Locus-Specific Transcript Clusters

**DOI:** 10.1371/journal.pone.0080961

**Published:** 2013-12-06

**Authors:** Matteo Chiara, David S. Horner, Alberto Spada

**Affiliations:** 1 Dipartimento di Bioscienze, Università degli Studi di Milano, Milano, Italia; 2 Dipartimento Di Scienze Agrarie E Ambientali - Produzione, Territorio, Agroenergia, Università degli Studi di Milano, Milano, Italia; University of North Carolina at Charlotte, United States of America

## Abstract

*De novo* transcriptome characterization from Next Generation Sequencing data has become an important approach in the study of non-model plants. Despite notable advances in the assembly of short reads, the clustering of transcripts into unigene-like (locus-specific) clusters remains a somewhat neglected subject. Indeed, closely related paralogous transcripts are often merged into single clusters by current approaches. Here, a novel heuristic method for locus-specific clustering is compared to that implemented in the *de novo* assembler Oases, using the same initial transcript collections, derived from *Arabidopsis thaliana* and the developmental model *Streptocarpus rexii*. We show that the proposed approach improves cluster specificity in the *A. thaliana* dataset for which the reference genome is available. Furthermore, for the *S. rexii* data our filtered transcript collection matches a larger number of distinct annotated loci in reference genomes than the Oases set, while containing a reduced overall number of loci. A detailed discussion of advantages and limitations of our approach in processing *de novo* transcriptome reconstructions is presented. The proposed method should be widely applicable to other organisms, irrespective of the transcript assembly method employed. The *S. rexii* transcriptome is available as a sophisticated and augmented publicly available online database.

## Introduction

The molecular mechanisms that underlie the generation and diversification of evolutionary novelties is one of the key issues in developmental biology [1]. In this respect, the genus *Streptocarpus* Lindl, which includes caulescent species that produce leaves from a conventional shoot apical meristem (SAM) as well as acaulescent species - both unifoliate (possessing only a single leaf throughout the individual’s life), and rosulate such as *Streptocarpus rexii* (producing several leaves arranged in a more or less irregular rosette) - is of particular interest. These acaulescent forms lack an orthodox SAM and therefore have no typical vegetative stem. The vegetative plant body of acaulescent *Streptocarpus* species consists of reiterated units composed of a stalk-region, termed petiolode because it possesses characteristics of both shoot and petiole, and a lamina, together termed phyllomorph. Continued growth of these structures is orchestrated from a nexus of three meristems at the junction of the lamina and petiolode: (a) the basal meristem which provides the growth of the lamina; (b) the petiolode meristem, involved in the growth of the midrib and elongation of the petiolode; (c) the *groove* meristem which provides the growth of new phyllomorphs and inflorescences. The unique morphologies observed within *Streptocarpus* are intrinsically linked to the interplay of these three meristems, which interact even at the seedling stage, and are responsible for the great vegetative diversity in the genus [2]. *Streptocarpus*, and other Old World Gesneriaceae, also exhibit anisocotyly, the unequal development of cotyledons after germination whereby the first phyllomorph is derived from the continued growth of one of the two cotyledons to form the macrocotyledon (cotyledonary phyllomorph), from the activity of the basal meristem [3].

Current genetic resources for *S. rexii* are scarce; molecular sequence data is extremely limited and no reliable and efficient genetic transformation protocols have been developed. Even information regarding its genome size is limited, although a correlation between aneuploidy and developmental patterns within the genus has been suggested [4]. The availability of an extensive sequence resource would greatly facilitate gene expression studies using realtimePCR and *in-situ* hybridization in crosses and hybrids which can show phenotypic variations.

The advent of Next Generation Sequencing (NGS) technologies has revolutionized genomics and functional genomics of both model and non-model species. For the latter category, sequencing and *de novo* assembly of the complement of expressed genes (the transcriptome) represents an attractive and cost-efficient alternative to whole genome sequencing to provide broad resources to assist in genetic and functional studies [5].

Typically, tens, or even hundreds of millions of short reads derived from cDNA are generated by high throughput sequencing technologies such as the Illumina platform and are assembled into putative transcripts without the use of a reference genome sequence.

De Bruijn Graph (DBG) based assemblers such as Velvet [6] exploit the concept of frequency and overlap of sequence strings of length “K” (Kmers) and are routinely used in the *de novo* reconstruction of genomes from short sequence reads. The optimal “Kmer” length for graph building is influenced by the depth of read coverage. However, the wide range of expression levels of different genes can limit the efficacy of DBG assemblers in *de novo* transcriptome assembly. Current transcriptome assemblers such as Oases [7] and TransAbyss [8], attempt to circumvent this problem by merging assemblies made using different Kmer values into a single, more complete assembly. While non-trivial, this final assembly step can increase the sensitivity of transcriptome reconstruction, facilitating the reconstruction of transcripts across a wide range of expression levels, it tends to lead to the generation of more redundant transcripts, and transcript variants [9].

Oases also attempts to organize sequences into loci or Locus-Specific Transcript Clusters (LSTCs). The method employed is analogous to the scaffolding of genomic contigs, and, in simple terms assumes that contigs (candidate exons) linked by paired end reads (or overlapped by single reads) should derive from the same genomic locus. However, in practice this is likely to merge transcripts from closely related paralogous genes into single loci [7], particularly for transcripts expressed at low levels. Despite this limitation, many studies performing *de novo* transcriptome assembly choose to select a single representative transcript from each Oases locus for downstream applications, risking the underestimation of gene diversity and number of true loci identified by the assembler.

A near optimal method for LSTC construction would consist of performing all possible pairwise alignments between reconstructed transcripts, using suitable overlap and identity cutoffs and hierarchical clustering steps to assemble final LTSCs. However, the computational demands of such an approach would be extremely high. A more feasible approximation of this strategy is to estimate overlap and identity cutoffs for clustering from distributions derived from within improved initial clusters. Indeed, pairwise alignment can reasonably be substituted with a Blast step, considerably reducing computational costs.

Here, we demonstrate the efficacy of the proposed transcript clustering approach using a publicly available RNA-seq dataset from *Arabidopsis thaliana* where a high quality reference genome and annotation is available. We provide simple metrics to evaluate the impact of the method when a reference genome is not available and apply the clustering strategy to transcripts reconstructed from a novel high throughput RNA sequencing dataset from *S. rexii*. We show that our approach notably improved the coherence of LSTCs with respect to the initial assembly and that a relevant proportion of genes are represented in the reconstructed transcriptome. The *S. rexii* transcriptome database is equipped with a series of tools to facilitate downstream analyses and is available at http://www.beaconlab.it/angeldust.

## Methods

### Arabidopsis thaliana RNA-seq Data

Raw data from an *Arabidopsis thaliana* RNA-seq experiment performed on leaf tissue (2×90 nt paired end sequence data from a HiSeq2000 instrument) was downloaded from http://www.ncbi.nlm.nih.gov/sra/?term=SRR764885.

### Sequence Preprocessing and Initial Transcriptome Reconstruction

A custom script implementing strict quality filters based on the provided base call quality scores was used to trim sequence reads prior to assembly. Reads were iteratively trimmed from the 3′ end until all of the following conditions were satisfied:

1. the median quality score (Qscore) of upstream bases was > = 25
2. less than 5 bases with Qscore < = 10 and less than 10 bases with Qscore < = 20 were present in the upstream sequence
3. the cumulative error probability in the upstream region was below 1E^−2^.

Only reads longer than 60 nt in length after trimming were used in assembly.

The Oases [7] pipeline was employed for initial transcriptome assembly. To ascertain optimal parameters for assembly, exploratory analyses were performed using 20% of the data with different ranges of Kmer values.

### Reference Database

A local Blast database containing all reference peptide sequences from *Arabidopsis thaliana*, (TAIR 10) [10], *Solanum Lycopersicum* (Solanum genome browser) [11], *Mimulus guttatus*, *Glycine max* and *Oryza sativa* japonica (all from Phytozome) [12] - for a total of 212,550 peptides was created. Sequences were associated with GO terms and Plaza [13] family homology and orthology terms.

A total of 255,990 cDNA and EST sequences from the same species were recovered from the TIGR gene index database (http://compbio.dfci.harvard.edu/tgi/).

### Clustering and Functional Annotation

The *ad-hoc* heuristic strategy developed to cluster transcripts into putative LSTCs was designed to provide a computationally tractable approximation of an all against all exhaustive pairwise alignment approach and can be described in three steps.

### Step 1: Coarse Clustering

The Cd-Hit-EST software [14] was used to sort the transcripts from within each Oases locus into “secondary loci”, within which all sequences showed at least 85% overall identity to the longest transcript in the cluster. Sequences representing perfect subsequences of other transcripts were discarded. This step splits many Oases loci into distinct secondary loci.

### Step 2: Annotation and Annotation-based Clustering

The longest transcript from each secondary locus was used in BlastX [15] similarity searches (Blosum80 scoring matrix) against an appropriate section of the reference database (see results) using BlastX. Best hits with a bitscore over 85 and an evalue < = 1E^−20^ were used to assign “best hit” annotations for each species to secondary loci.

Secondary loci with identical cross species profiles of best hit annotation were merged into “tertiary loci”, and an analogous annotation and clustering step was performed for non-annotated secondary clusters using BlastN searches of ESTs and cDNAs from the same combination of species. Finally, all secondary clusters lacking Blast annotation were used to search the NCBI non-redundant nucleotide collection. Transcripts giving significant (evalue< = 10E^−5^) against non-plant species were marked as potential contaminants.

### Step 3: Generation of Definitive LSTCs

All transcripts within each “tertiary locus” were aligned with each other using BlastN and custom scripts were used to define distributions of identity and overlap of pairs of transcripts within tertiary loci. The rightmost 1% tail of these distributions was established and the corresponding values used as estimates of the minimum identity and overlap thresholds for subsequent, final clustering step.

All against all Blast searches were performed for all transcripts and agglomerative hierarchical clustering was used to assemble definitive LSTCs wherein linked combinations of matches gave alignments surpassing the aforementioned identity and overlap thresholds.

From each LSTC, a final representative sequence was chosen; for annotated LSTCs this was the sequence giving the highest scoring BlastX match against the original peptide database (for LSTCs where more than one transcript gave similar highest scores, the shorter isoform was selected), for unannotated LSTCs, the longest transcript model was chosen.

### Functional Annotation and Gene Family Assignment

When available, functional annotation (GO terms) were inherited directly from peptide sequence to which the representative sequence gave the best hit. To assign definitive LSTCs to putative gene families, the entire Plaza [13] fam homology and orthology databases were used and hom-fam and ort-fam terms were inherited from best Blast hits.

### Estimation of Intronic and UTR Content of Transcripts

Regions likely corresponding to retained introns were identified from “confidently annotated representative sequences” (those with >75% coverage of a unique protein from a reference species) - assuming overall co-linearity of CDSs and conservation of CDS length - using custom scripts to parse Blast outputs. Putative UTRs are defined as transcript sequences upstream and downstream of terminal CDS derived HSPs (excluding the appropriate number of bases to account for unaligned N- and C-terminal amino acids). Putative retained introns are defined as regions within the CDS alignment (also accounting for poorly conserved, non-aligned residues) in excess of the expected CDS length and not providing significant Blast matches to annotated coding regions.

### *S. rexii* Plant Material, Growth Conditions and Tissue Preparation

Plants and seed materials of *Streptocarpus rexii* Lindl. (Gesneriaceae; Lindley 1828), RBGE accession number 20030814 (Tsitsikamma, Cape Province, SA), came from a population derived from 3 original plants from the living research collection held at the Royal Botanic Garden, Edinburgh and grown in glasshouses at the Botanical Garden of the University of Milan. Voucher specimens are deposited at the herbarium at the Royal Botanic Garden Edinburgh.

Seedlings at different developmental stages (from unfolded cotyledon to late anysocotyledony) - around 10,000 seedlings in total, as well as the basal parts of phyllomorphs (20 plants) were used for RNA extraction.

### Library Preparation and Sequencing

Total RNA was extracted using the SIGMA Spectrum Plant Total RNA Kit (Sigma-Aldrich). Sequencing libraries, enriched in PolyA+ RNA fraction, were constructed using the TruSeq RNA-seq sample prep kit from Illumina (Illumina, Inc., CA, USA) according to manufacturer’s instructions. mRNA-seq libraries were loaded into a single lane of a flowcell v. 3 and sequenced in paired-end 100 bp set-up with a HiSeq2000 sequencer by IGA Technology Services, Udine, Italy. Sequence data are deposited in the NCBI Short Read Archive in BioProject PRJNA189663.

### Database Structure and Implementation

The web interface was implemented using the RoR (Ruby on Rails) web development framework (http://rubyonrails.org/), while the core database is implemented in mySQL (www.mysql.com). Style sheets and css are from the twitter-bootstrap project (http://twitter.github.com/bootstrap/). Blast search functionality is provided through a customization of the sequence-server project (http://www.sequenceserver.com/). Primer degin and *in silico* PCR tools are provided through customization of the web interfaces of primer3 [16] and MFE primer [17] applications.

## Results

### A Novel Heuristic for the Construction of Locus-Specific Transcript Clusters (LSTCs)

Oases “loci” (groups of sequences expected to derive from individual genomic loci) are reconstructed by searching for highly interlinked paths in assembly graphs. However, this approach is prone to merge transcripts from closely related, or poorly expressed paralogous genes into single loci [7].

**Figure 1:**
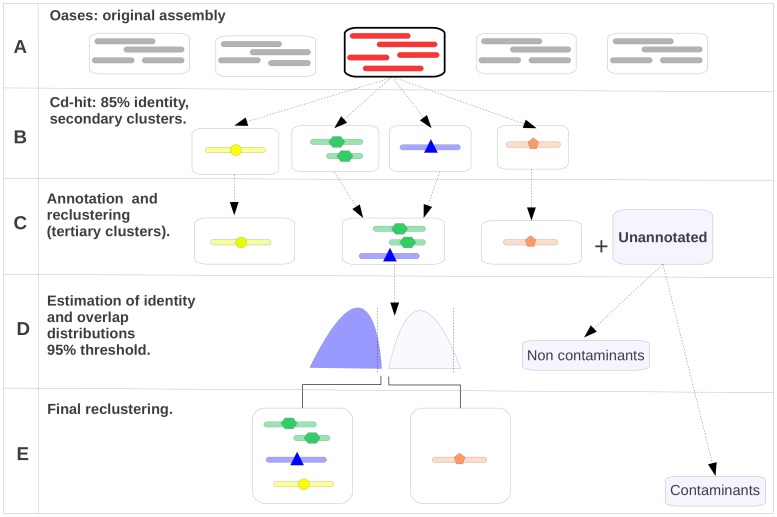
Schematic representation of strategy for re-clustering of transcript loci generated by Oases. The figure presents a simple flow diagram illustrating the strategy used to re-assemble clusters of transcripts into LSTCs. As an example, we consider a single hypothetical Oases locus. A) An original Oases locus. B) Secondary loci are constructed by Cd-Hit-EST and defined such that within each secondary cluster, all transcripts must show 85% identity to the longest transcript. Thus the original Oases locus is split into 4 secondary loci. C) Representative sequences from each cluster are assigned “Blast annotations” and clusters with identical blast annotation profiles across proteomes used in annotation are merged into tertiary loci. D) All against all Blastn searches within clusters are used to estimate distributions of overlap and identity of transcripts within tertiary loci. E) All against all blast searches of transcripts in tertiary loci are used to merge clusters into LSTCs where overlaps and identities exceed cutoffs obtained in the previous step.

A theoretically sound strategy for improved construction of LSTCs would be to align all pairs of transcripts, using dynamic programming with suitable parameters, to detect significant overlaps and then to use agglomerative hierarchical clustering to assemble final clusters. Such an approach implies an enormous number of pairwise alignments and a significant effort to estimate suitable overlap and identity thresholds for cluster formation. Here, overlap and identity cutoffs for clustering are estimated from distributions derived from alignments within existing loci and pairwise alignment is substituted with Blast searches, considerably reducing computational costs. Indeed, two simple initial steps are applied in an attempt to improve the integrity of clusters used for parameter estimation. The approach adopted is described in detail in materials and methods and illustrated in Figure 1.

Briefly, initial loci (Figure 1A) can be subdivided into “secondary loci” (Figure 1B) wherein all members show at least 85% identity to the longest representative sequence (some correctly grouped transcripts are divided in this step, along with many transcripts that might have been over-clustered by the assembler). The 85% overall identity criterion is implemented to overcome differential intron retention in assembled transcripts from individual gene loci.

Longest representatives of each secondary locus are used in similarity searches of the proteomes of 5 representative plant species (see materials and methods) and satisfactory best match annotations from at least one species are assigned, where possible, to clusters.

Where more than one secondary locus gives identical cross-species profiles of best hits (Figure 1C), the associated secondary clusters are merged into “tertiary loci”. This step attempts to connect discontiguous fragments of transcripts from shared loci.

Representative sequences of loci that do not provide a significant hit against tested plant proteomes can additionally be used to search EST and genomic sequences of the same organisms using BlastN, and an analogous tertiary clustering – based on shared hits can be performed. Searches of plant tRNA and rRNA databases as well as of non-plant sequences can be used to identify contaminants and other non desirable sequences.

At this point, BlastN-derived alignments of sequences within the “improved” tertiary clusters can be used to model distributions of overlap and identity expected for transcripts and transcript fragments that derive from the same genomic locus (Figure 1D). The rightmost tails of such distributions are used to determine cutoff values of overlap and identity to be used to allow hierarchical agglomerative clustering of transcripts into final LSTCs through all against all Blast searches (Figure 1E). It should be noted that the false discovery rate indicated from the choice of the cutoffs (1% is the arbitrarily chosen value) will be a radical overestimate of the real FDR in the final clustering phase as the majority of the alignments used to establish the cutoff derive from correctly clustered sequences, or highly related paralogs.

From the final LSTCs, the shortest transcript generating the best hit to the reference transcriptome/proteome database will be selected as a representative transcript and gene family and GO terms for the LSTC will be inherited from its best hits. This selection is designed to minimize the use of transcripts with retained introns as representative sequences. For LSTCs without detectable similarity to annotated transcripts from reference genomes, the longest transcript might be selected as a representative sequence.

### Evaluation of Improvement in Transcript Clustering

We propose several simple approaches for the evaluation of the quality (and improvement in the quality) of clusters of transcripts in the absence of a reference genomic sequence, and in subsequent sections, demonstrate their efficacy by employing them in a control scenario (using *de novo* assembled and clustered transcriptome data from *Arabidopsis thaliana*).

Firstly, the number of cluster representative transcripts giving significant best Blast hits to a homologous protein encoded by a related reference genome can be compared to the number of distinct reference proteins identified. Assuming an essentially similar proteome composition between related species, a ratio close to 1 suggests minimal fragmentation of clusters. Of course high numbers of distinct reference proteins identified also hint at a broad coverage of the sampled transcriptome.

Secondly, cluster representative sequences can be simply assigned to gene families by inheriting information on homologous sequences from the Plaza [13] gene family database. Assuming generally similar gene family sizes between related organisms, high Pearson correlation coefficients between gene family sizes are consistent with both complete and well clustered transcript data. It is straightforward to estimate the significance of differences in gene family size correlation values between clustered assemblies. Such tests can be performed independently for gene families whose sizes increase or decrease.

Thirdly, the presence and cluster distribution of genes known to be present as single copy conserved orthologs in related genomes can be evaluated. The proportion of such genes that are included as cluster representative sequences and the proportion matching 2 or more distinct representative sequences will hint at the completeness of the transcript collection and the fragmentation of clusters.

### Assembly and Re-clustering of *Arabidopsis thaliana* Transcriptome Data

To evaluate the proposed transcript clustering strategy, a publicly available wild-type *A. thaliana* (Col) RNA-seq dataset generated using a HiSeq2000 instrument (SRR764885) was first analyzed. The nature of the library, numbers of reads, results of quality trimming and characteristics of the initial transcriptome assembly obtained using Oases (using a kmer range of 25–39– established as appropriate from preliminary reconstructions with 20% of the data) are shown in Table 1. Cd-Hit-EST subclustering reorganized the Oases loci into 39,816 secondary loci. The annotation-based clustering step (excluding *A. thaliana* sequences from the reference database), permitted the generation of 26,060 annotated tertiary loci and 13,810 unannotated tertiary loci. 280 unannotated representative sequences gave significant hits to plant tRNA/rRNA or representative non-plant sequences and their associated LSTCs were discarded from the dataset.

**Table 1:** Summary of assembly statistics.

	*A. thaliana*		*S. rexii*	
Raw data^1^				
Read type	2*90 nt paired end		2*100 nt paired end	
Total pairs	25,748,028		92,353,379	
Pairs after trimming	20,015,515		64,243,530	
Unpaired reads	1,503,212		2,503,141	
Total nt	4,171,180,536		11,134,167,085	
**Assembly and annotation** 2				
Original oases loci	33,272		44,303	
Original oases transcripts	77,980		843,303	
Secondary loci(CD-HIT-EST)	39,816		239,406	
**Tertiary loci** 3				
Annotated	26,060		35,704	
Unannotated	13,810		38,588	
Contaminants	280		9,503	
Shorter than 350 annotated	208		3,504	
Shorter than 350 unannotated	13,410		15,512	
**Final assembly** **(filtered)** 4	**Oases**	**LSTCs**	**Oases**	**LSTCs**
Annotated	25,807	23,203	24,832	20,252
Unannotated	683	400	8,281	6,895
N50 representative	1,542	1,556	2,201	2,252
N50 confidentlyannotated	1,683	1,694	2,803	2,728
**Read mapping stats** 5				
To LSTC representative	17,176,209		29,401,372	
To LSTC complete	18,576,209		60,334,068	
To contaminants	950,203		11,303,201	

1 Characteristics of sequence data used in the assembly.

2 Results of the original assembly by Oases and initial clustering by CD-HIT-EST.

3 Statistics regarding representative sequences of tertiary loci.

4 Statisitics regarding LSTCs and Filtered Oases clusters.

5 Number of reads mapping to the reference transcript collections.

The length distributions of representative sequences from annotated and un-annotated tertiary loci differed greatly: less than 1% of the former and 97% of the latter were shorter than 350 nt. Mapping of the 13,410 unannotated short transcripts to the *A. thaliana* genome sequence revealed some striking patterns. Indeed, 7,950 mapped to annotated CDS regions and c. another 3,500 to annotated UTRs, c. 1,100 were in intergenic locations while 850 did not map in a coherent manner to the genome. 7,445 mapped to genes which that had already been identified by annotated transcripts and indeed, less than 500 genes were identified only by short transcripts. Thus, while 33.6% of clusters comprised of only short transcripts, they were responsible for the identification of less than 3% of the genes matched by tertiary cluster representative sequences. Over 88% of the genes identified only by short transcripts are *A. thaliana*-specific with respect to the other proteomes employed in annotation. Accordingly, to reduce computational complexity of the final clustering step, all transcripts of less than 350 nt in length were excluded from the final LSTC construction step which generated 23203 and 400 annotated and unannotated LSTCs respectively.

### Evaluation of Assembly and LSTCs

The N50 value for the representative sequences from the *A. thaliana* LSTC representative transcripts was 1,556 (rising to 1,694 for “confidently annotated” LSTCs – those whose representative sequence gave a match spanning >75% of a homologous CDS from another species). These numbers closely resemble those from the Oases loci filtered by the same size and contaminant criteria (1,542 and 1,683), and correspond well with expected values (N50(cds) = 1,536, N50(mRNA with annotated UTR) = 1,918), suggesting that the initial transcript assembly is of good quality. The proportions of initial reads mapping to representative transcripts and all transcripts are shown in Table 1 and indicate comprehensive incorporation of the data into the reconstructed transcriptome. Interestingly, of 133 potentially chimeric transcripts (those providing matches to multiple reference sequence from distinct gene families), 92% correspond to loci with less than 1.5 Kb between them on the arabidopsis genome, suggesting that incomplete gene annotation or naturally overlapping transcripts (rather than over-assembly) are the principal causes of this phenomenon.

23,603 LSTC representative sequences were mapped to the *A. thaliana* genome. 23,203 of these overlapped 16,900 distinct annotated loci (mean 1.37 transcripts for each identified annotated locus). When the Oases loci (filtered for contaminants and short, unannotated sequences) were subjected to the same test, 25,807 representative transcripts matched 16,128 distinct genes (ratio = ∼1.58), indicating that our clustering strategy increased the coherency of LSTCs both by separating incorrect clusters and merging redundant clusters. The results of a similar experiment, using best significant hits to the proteomes of the 4 other reference species, are shown in Table 2, indicating the expected trends of improvement in gene discovery and reduction of cluster redundancy (p = 0.02 (t-test)), are maintained when similarity - rather than identity to the genome of origin – is used as a proxy for gene discovery.

**Table 2:** Numbers of distinct matched genes from reference genomes and mean numbers of best matching representative transcripts for Oases loci and LSTCs.

	*A. thaliana*				*S. rexii*			
	Oases		LSTCs		Oases		LSTCs	
Species (total loci)^1^	match^2^	mean^3^	match^2^	mean^3^	match^2^	mean^3^	match^2^	mean^3^
*O. sativa (55,851)*	10,394	1.95	10,961	1.67	11,253	1.94	11,554	1.5
*A. thaliana* (27,416)	16,128	1.58	16,900	1.37	12,109	1.97	12,427	1.45
*G. max* (46,367)	13,392	1.65	14,000	1.43	14,058	1.73	14,428	1.29
*S. lycopersicum* (34,727)	11,348	1.92	11,960	1.66	12,798	2.02	13,106	1.44
*M. guttatus* (28,282)	11,621	1.89	12,101	1.64	12,936	2.03	13,317	1.44

1 loci in the reference proteome.

2 distinct genomic loci matched by the transcript collections.

3 average number of loci in the collection per matched genomic locus.

In total, 4,351 Oases loci were modified by the re-clustering, giving rise to 2,572 LSTCs. Of the original Oases loci, 503 (11%) were incoherent (contained transcripts mapping to more than one annotated genomic locus). Of the resulting LSTCs, 43 (1.6%) were incoherent.

Pearson correlation coefficients of gene family size between the genomic annotations and those associated with either Oases loci or re-clustered LSTCs (as inherited from best matches for representative sequences) also improved from 0.88 to 0.91 (from 0.929 to 0.932 for families whose dimensions increased after re-clustering and from 0.888 to 0.910 for families whose dimensions decreased after re-clustering (Table S1). Table 3 shows that equivalent coefficients of correlation based on similarity matches, rather than assignment to the genome of origin, follow the expected pattern - supporting the validity of this metric in the comparison of clustering strategies.

**Table 3:** Comparison between gene family size correlation coefficients between LSTC collection and the filtered Oases collection.

	*A. thaliana*		*S. rexii*	
	Oases^1^	LSTCs^2^	Oases^1^	LSTCs^2^
*A. lyrata*	0.87	0.91	0.75	0.84
*A. thaliana*	0.88	0.91	0.76	0.85
*B. dystachion*	0.8	0.83	0.75	0.85
*C. papaya*	0.86	0.85	0.79	0.9
*F. vesca*	0.85	0.87	0.69	0.78
*G. max*	0.86	0.9	0.78	0.88
*L. japonicus*	0.87	0.9	0.76	0.85
*M. domestica*	0.84	0.87	0.67	0.76
*M. esculenta*	0.84	0.88	0.79	0.88
*M. truncatula*	0.67	0.7	0.54	0.61
*O. sativa*	0.75	0.78	0.68	0.77
*P. trichocarpa*	0.83	0.88	0.69	0.89
*R. communis*	0.86	0.88	0.78	0.85
*S. bicolor*	0.75	0.78	0.63	0.72
*T. cacao*	0.82	0.83	0.71	0.8
*V. vinifera*	0.82	0.84	0.73	0.82
*Z. mays*	0.79	0.83	0.78	0.88

1 correlation coefficients between the size of gene families in the Plaza hom fam database for different reference species and that inferred from the original Oases assembly+filtering.

2 correlation coefficients between the size of gene families in the Plaza hom fam database for different reference species and that inferred from our LSTC collection.

### *Streptocarpus rexii* Lindl. Transcriptome Assembly and Re-clustering

Deep sequencing of *Streptocarpus rexii* cDNA (see materials and methods) generated 92,353,379 pairs of 100 nt reads. Stringent preprocessing and read trimming steps allowed the recovery of 64,243,530 paired ends, with average length of 85.5 nt. Initial *de novo* transcript assembly using Oases, and preliminary reconstructions with 20% of the data indicated that Kmer values in the range of 21–39 gave best results (based on the N50 of assembled transcripts and proportions of reads mapping back to the assembly). Given that empirical observations from initial assemblies, as well as the nature of the sequencing library construction protocol employed, suggested that insert sizes were rather heterogeneous, insert size parameters were not set and scaffolding options in Oases were disabled for transcriptome assembly.

Information regarding the output of Oases and the steps involved in the generation of LSTCs are presented in Table 1 and show that while Oases generated a comparable number of loci to the *A. thaliana* experiment, an order of magnitude more initial transcripts were recovered. This is likely to be due in part to the absence of the scaffolding step, but also to the fact that mixed tissue samples and over three times as many initial reads were employed - allowing the recovery of a larger number of low abundance transcript variants. The number of secondary loci generated by Cd-Hit filtering is over 6 times greater than observed in the *A. thaliana* experiment, although the annotation based clustering resulted in less than twice as many tertiary clusters as observed in the *A thaliana* test. The observation that a higher proportion of tertiary clusters remained unannotated by similarity to reference proteomes might in part derive from the phylogenetic distance between *Streptocarpus* and other well annotated genomes, but for the most part was caused by the fact that representative sequences of 9,503 unannotated tertiary loci gave significant hits against plant t/rRNA genes and sequences of non-plant origin (predominantly ribosomal RNA sequences of fungi of the genus *Fusarium* and bacteria of the genus *Bacillus* as well as some insects). Such clusters were treated as contaminants and excluded from further analyses. Interestingly, most of the rRNA contaminants (6,889) were grouped by Oases into a single original locus. Very few unnanotated representative transcripts (<20), gave best BlastX hits against non-plant proteins, and the significance of such hits was universally marginal. 40.2% of representative sequences from unannotated loci were less than 350 nt in length (7.31% for annotated sequences). As observed in the *A. thaliana* dataset, a large proportion (88.9%) of the annotated short transcripts gave best matches to reference database sequences also identified by other longer transcripts. Given the low rate of gene discovery by short transcripts in the *A. thaliana* experiment, it was decided to exclude all sequences shorter than 350 nt from the main transcript collection (although they are available in a distinct section of the final database - see below).

Distributions of identity and overlap were estimated for matches between sequences within tertiary loci and the rightmost 1% tail of overlap and identity distributions in such comparisons were calculated (95% identity, 65% overlap). After the final alignment and agglomerative hierarchical clustering steps, the definitive transcript collection contained 20,252 annotated and 6,895 un-annotated LSTCs.

The 44,303 original Oases loci were treated with the same filters as employed for our re-clustered data, 1,680 clusters were discarded as contaminants, and 7,509 loci had representative sequences less than 350 bases in length, leaving 26,832 annotated and 8,281 non annotated loci.

### Characteristics of the *S. rexii* Transcriptome Assembly

88% of the final *S. rexii* LSTCs contain < = 15 transcripts while 2.5% contain more than 30 alternative or partial transcripts (0.24% with more than 100 transcripts). Proportions of initial reads mapping to various components of the LSTCs and tertiary clusters are shown in Table 1 and indicate notable differences from the *A. thaliana* data. In particular, a much higher proportion of reads map to contaminant tertiary clusters (17% vs 4.4%), while a lower proportion of reads map to representative transcripts.

The N50 value obtained with LSTC representative transcripts was 2,252 (rising to 2,728 for “confidently annotated representative transcripts”) - rather higher than expected, the equivalent value for transcripts with annotated UTRs in the genomes used for annotation ranging from 1,658 nt (Mimulus) –2,292 nt (rice). Similar values were observed for filtered Oases clusters, suggesting that the N50 observed derives from the assembly rather than clustering, and consistent with the fact that no additional assembly was performed. Several possible factors could explain the anomalous N50 values: inherently longer CDS or UTRs in *S. rexii*; over-assembly of transcripts by Oases; or potentially a high level of intron-retention in the reconstructed transcripts.

A preponderance of chimeric transcripts is excluded by analyses showing that only 136 of nearly 27,000 annotated LSTCs generate Blast hits against more than one distinct protein family from reference species. Exclusion of these transcripts from N50 calculations reduces the global N50 from 2,252 to 2,249 (2,728 to 2,703 for representative transcripts), confirming that the presence of chimeric transcripts is not a major cause of observed N50 values.

The potential impact of UTR length or intron retention on N50 values was investigated for over 2,800 “confidently annotated” (CA) representative transcripts with unambiguously identifiable *A. thaliana* orthologs (see materials and methods). The proportion of transcripts represented by putative UTRs is essentially similar for both datasets (Figure S1a), while the proportion of putatively retained intronic sequence in reconstructed *S. rexii* transcripts is almost double that in *A. thaliana* (Figure S1b). Table 4 shows that exclusion of the putative intronic regions, allows recovery of CDS and transcript N50 values within the expected range. The G+C content of putative introns (Table 5) shows a highly significant (p = 3.3E^−64^) difference to that of exonic regions in accord with the general pattern in plants (higher GC in coding regions) and consistent with a parallel analysis of retained introns identified from genomic annotation in the *A. thaliana* data. Additionally, analysis of read maps shows that the mean RPKM associated with putative exonic regions was 4.42 times higher than of putatively retained introns. For the *A. thaliana* data (Table 5) the ratio of exonic to retained intron RPKM was higher, consistent with less frequent intron retention. Taken together, these data indicate that widespread intron retention, or a high proportion of incompletely processed transcripts in the RNA used for library construction, underlie the unexpectedly high N50 values for the reconstructed *S. rexii* transcriptome (see discussion).

**Table 4:** Impact of inferred UTRs and putative retained introns on N50 of LSTC representative transcripts.

		InferredN50	N50annotation^1^	N50transcripts
*A.thaliana*	**CDS** 2	1,481	1,536	1,650
	**CDS+UTR** 3	1,624	1,815	
*S. rexii*	**CDS** 2	1,464	1,636	2,300
	**CDS+UTR** 3	1,741	1,841	

1 N50 of *A. thaliana* orthologs.

2 N50 of CDS, excluding inferred UTR and putative retained introns.

3 N50 of CDS and UTR, excluding putative retained introns.

**Table 5:** Mean GC content and read coverage of inferred CDS and putative retained introns.

	CDS^1^	Intronic^2^	Pvalue^3^	Exon/Intron fold^4^
*A. thaliana*	43.9 (3.44)	37.1 (2.88)	2.90E-61	8.72
*S. rexii*	46.18 (4.81)	39.67 (3.04)	3.30E-64	4.42

1 mean G+C% (and standard deviation) in inferred CDS.

2 mean G+C% (and standard deviation) in inferred intronic regions.

3 Pvalue for difference of GC content between CDS and putative retained introns (t-test).

4 ratio between the RPKM calculated on the inferred exonic and intronic regions.

4.5% of confidently annotated LSTCs gave satisfactory (evalue< = 10E^−20^) matches only against genes from *M. guttatus*, while 1.3% recovered such hits only from *S. lycopersicum*. All other species gave low frequencies (<1%) of such “unique” matches. Between 17% (*O. sativa*) and 47% (*M. guttatus*) of the proteomes of reference species were recovered in high significance Blast hits using our LSTC collection as probes. Both of these observations are consistent with phylogenetic considerations (*S. rexii*, *S. lycopersicum* and *M. guttatus* are all members of the Lamiales).

### Evaluation of Clustering and Annotation Steps

Mean numbers of LSTC representative sequences matching each protein recovered from reference genomes vary between 1.29–1.5. However, for the size and contaminant filtered Oases loci, the same statistic ranges between 1.73 and 2 (p = 5E^−04^, t-test), indicating that our strategy successfully merged a notable number of Oases loci while splitting over-clustered loci - in line with the observations for the *A. thaliana* dataset (Table 2).

The impact of the novel clustering strategy on gene family size correlations is shown in Table 3 (full data in Table S1). For 17 higher plant genomes supported in the Plaza database, mean gene family size correlations increase significantly (p = 3E^−04^).

COSII markers [18] are PCR-based markers developed from a set of single-copy conserved orthologous genes (COSII genes) in Asterid species. Each COSII gene (representing a group of Asterid unigenes) matches only one single-copy Arabidopsis gene. COSII genes were used to interrogate the Oases and LSTC representative sequences and the number of distinct loci/LSTC representative sequences providing significant hits for each COSII gene was recorded. Table 6 shows that, for both the *A. thaliana* and *S. rexii* datasets, the fragmentation of COSII genes is notably reduced by the re-clustering method. Indeed, in both cases, nearly 200 additional COSII genes are identified as representative transcripts after re-clustering.

**Table 6:** COSII gene discovery and cluster fragmentation.

Clusters per COSII^1^	*A. thaliana*		*S. rexii*	
	Oases	LSTC	Oases	LSTC
> = 4	98	65	141	75
3	115	83	184	107
2	423	494	298	344
1	1,789	1,990	1,699	1,992
absent	444	257	567	351

1 number of COSII genes matching the specified number of reference transcripts.

### A Next Generation Locus Database Unveiling the Streptocarpus Transcriptome (ANGeLDUST)

The *S. rexii* transcriptome has been incorporated into an openly available online database with a user-friendly web interface. The sequence data is organized into the LSTCs outlined previously and representative sequences or the entire transcript collection (including or excluding short transcripts) can be interrogated through sequence similarity, gene names, Plaza gene family identifiers or GO terms. Individual sequences or alignments of entire LSTCs can be visualized or downloaded by users, and links to the Ensembl plant genome browser are available for reference sequences from genomes used in annotation. Furthermore, PCR primer design and “in-silico” electrophoresis tools are incorporated to facilitate the design of primers for “wet” experimental approaches.

Advanced functionalities permit the retrieval all the LSTCs assigned to a particular Plaza Homology or Orthology family and to compare the estimated of the family in *S. rexii* with that of other plants by the means of a direct link to the Plaza db. Links to download various sections of the dataset in fasta format are provided.

The ANGeLDUST database is available at http://www.beaconlab.it/angeldust.

## Discussion

The generation of transcript assemblies with multiple Kmer values can greatly enhance sensitivity across a wide range of expression levels and several strategies have been proposed to merge assemblies and reduce redundancy. Several studies merged distinct Kmer assemblies by eliminating contigs that are perfect subsequences of longer transcripts [19], [20]. Indeed, Oases itself also provides the possibility of merging distinct assemblies using a single Kmer approach [9]. Notwithstanding the exact approach employed for transcriptome reconstruction, few studies have explicitly addressed the accurate clustering of reconstructed contigs into LSTCs - required for the study of gene families, alternative splicing, the discovery of genetic markers etc. Oases uses reads shared between transcripts to propose LSTCs, while Surget-Groba et al. [19] used similarity to reference proteomes to assign transcripts to putative LSTCs – recognizing the dependence of their approach on comprehensive data from a closely related organism and the risk of merging transcripts from recently duplicated genes. The clustering strategy suggested here also uses similarity to reference sequences, but only as a step towards defining parameters for overlap-based clustering. Our approach is computationally tractable and has been shown to perform well using a control case where high quality genomic sequence and annotations are available. In addition it has been applied to improve a newly generated and assembled transcript collection for the developmental model *Streptocarpus rexii*, allowing the generation of the first extensive genetic resource for this fascinating plant.

A detailed comparison of the relative performance of transcriptome assembly tools is beyond the scope of the current work, indeed, published studies suggest that assemblers can show dataset and transcript expression level-specific relative performance advantages [7], [21]. Our method can easily be employed on the output of any assembler, including Trinity [22] which does not employ a multiple Kmer strategy (or indeed on conventional EST collections), and the metrics suggested for comparison of different locus/LSTC sets employed to chose a preferred final assembly.

Until now, many *de novo* transcriptome assembly studies have not fully exploited the capacities of available assemblers and have instead chosen to select a single Kmer assembly; typically that which yields the largest N50 value for assembled transcripts. A particular risk associated with this approach can be sub-optimal reconstruction of messages that are not best represented by the chosen Kmer. In the current study, the full Oases pipeline was employed, as the objective was to maximize the representation of *S. rexii* transcripts from mixed tissue samples. However, mapping of paired end reads to various assemblies obtained during calibration of the appropriate Kmer lengths revealed a highly heterogeneous size distribution of library inserts and accordingly, scaffolding options were disabled during the reconstruction of the *S. rexii* transcriptome. *A- posteriori* scaffolding steps obtained poor results (not shown), partly because of heterogeneous insert sizes and the expected heterogeneity of splicing isoforms and expression levels in mixed tissues. Indeed, this limitation in the assembly process likely contributes to the marginally improved apparent performance of the re-clustering procedure in *S. rexii* with respect to the *A. thaliana* experiment.

The observed high N50 value for the reconstructed *S. rexii* transcriptome was of particular interest. Thorough bioinformatics analyses strongly suggest that extensive intron retention in the mRNA used for sequencing underlies this phenomenon. Intron retention is known to be a widespread phenomenon in many higher plants (reviewed in [23]) and it is possible that it is particularly pervasive in *S. rexii*. However, the high levels of “contaminant” transcripts, particularly plant tRNA and rRNA also raise the possibility that a significant amount of incompletely processed (polyA-) mRNA was represented in the sequencing library, leading to identification of unspliced introns. Alternative, but perhaps less likely explanations for (apparent) intron retention might be defects in regulated RNA decay pathways or extensive antisense transcription. In any case, the widespread reconstruction of transcripts with retained introns seems not to be an inevitable feature of multi Kmer assemblies, as the phenomenon was considerably less evident with the *A. thaliana* data.

In addition to the observed intron retention, a number of other phenomena can lead to errors in transcript assembly and clustering. Firstly, when several real transcripts share stretches of identity that are longer than the Kmer size used, the initial assembly step can combine regions of close paralogs or alleles into single contigs that can be difficult to discriminate from the results of homologous recombination. This can be a particular problem in genomes exhibiting high levels of heterozygosity or in mixed populations of potentially out-breeding individuals as employed here (the material used for sequencing of the *S. rexii* transcriptome derived from three parental individuals). These considerations, along with authentic alternative splicing and intron retention are likely reflected in the relatively large mean size of LSTCs observed in the reconstructed *S. rexii* transcriptome.

Furthermore, particular combinations of biologically relevant alternative splicing patterns are difficult to recognize and some alternative splicing isoforms reconstructed may not be physiologically present. Additionally, some frameshift errors are expected, particularly for transcripts expressed at low levels and it is expected that some genes with low expression levels will be partially reconstructed or absent.

With respect to LSTC generation, a failure to merge loci where messages were fragmented and did not show extensive overlap is expected. Reduction of stringency of overlap and identity cutoffs in the final clustering step of our pathway will reduce the impact of this problem, but will also risk increasing the artifactual clustering of paralogs.

All of these considerations indicate that users of the *S. rexii* transcriptome database (and indeed of any *de novo* assembled transcriptome dataset) should carefully align individual gene sequences (and their complete LSTCs of origin) with homologous sequences from related organisms, to precisely identify likely retained introns and estimate phylogenetic relationships of individual sequences before selecting primers for amplification or regions to use a probes for expression analyses. Nevertheless, the wide gene and transcript sampling obtained and the frequent presence of assembled UTR sequences suggest that the ANGeLDUST website should represent a valuable resource for genetic, molecular and evolutionary studies in this intriguing organism.

## Supporting Information

Figure S1 **Box plots of percentages of representative transcripts corresponding to putative UTR and putative retained introns.** Figure S1a: box plot of percentage of transcripts corresponding to UTRs as estimated by parsing of Blast alignments with *A. thaliana* orthologs. Data are shown for 1896 (Ath) and 2904 (*S. rexii*) “confidently annotated” representative transcripts from LSTCs. Figure S1b: box plot of percentage of transcripts corresponding to putative retained introns as estimated by parsing of Blast alignments with *A. thaliana* orthologs. Data are shown for 1896 (Ath) and 2904 (*S. rexii*) “confidently annotated” representative transcripts from LSTCs.
(PDF)

Table S1 **Plaza homfam gene family sizes for a selection of completely sequenced plant genomes, and for transcriptomes reconstructed in the current study. Individual worksheets show results for complete transcriptome and gene families whose predicted dimensions either increased or decreased with the reclustering procedure described.** For each worksheet, Pearson correlation coefficients for Oases loci and reclustered LSTCs are shown.
(XLS)
