# Supplementary material for: De Novo Assembly of the Transcriptome of the Non-Model Plant Streptocarpus rexii Employing a Novel Heuristic to Recover Locus-Specific Transcript Clusters
Source: PLoS One. 2013 Dec 6;8(12):e80961. doi: 10.1371/journal.pone.0080961 (PMC3855653; doi:10.1371/journal.pone.0080961)

Supporting figure S1: Box plots of percentages of representative transcripts corresponding to putative UTR and putative retained introns.

Figure S1a: box plot of percentage of transcripts corresponding to UTRs as estimated by parsing of Blast alignments with *A. thaliana* orthologs. Data are shown for 1896 (Ath) and 2904 (*S.rexii*) “confidently annotated” representative transcripts from LSTCs

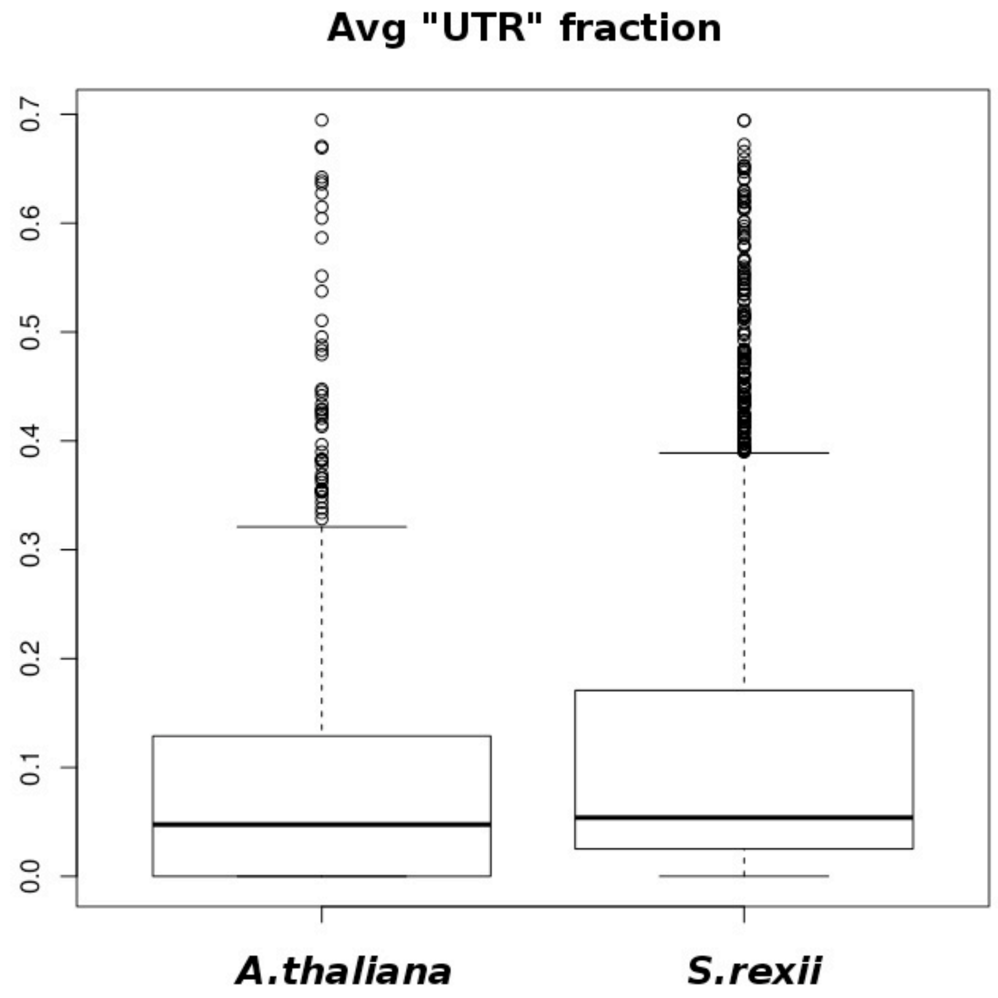

Figure S1b: box plot of percentage of transcripts corresponding to putative retained introns as estimated by parsing of Blast alignments with *A. thaliana* orthologs. Data are shown for 1896 (Ath) and 2904 (*S.rexii*) “confidently annotated” representative transcripts from LSTCs.

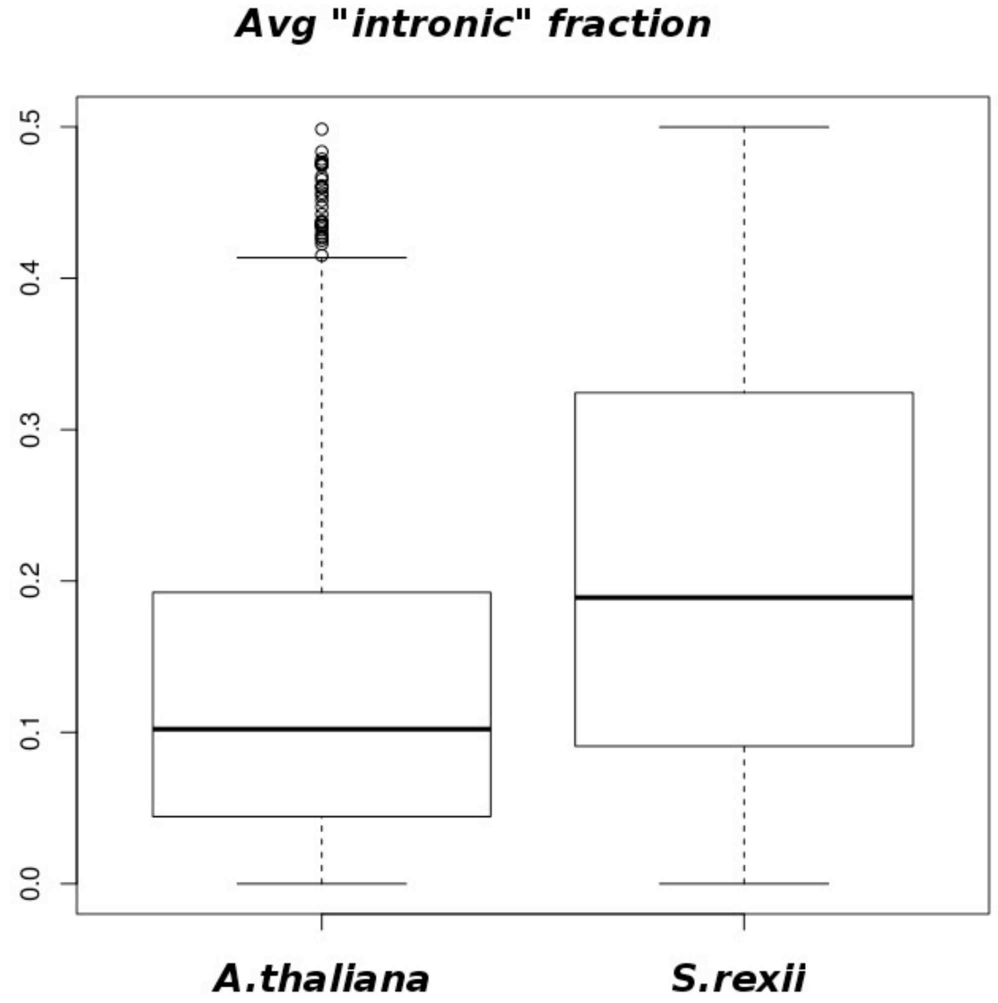

Supplement: Figure S1 — Box plots of percentages of representative transcripts corresponding to putative UTR and putative retained introns. Figure S1a: box plot of percentage of transcripts corresponding to UTRs as estimated by parsing of Blast alignments with A. thaliana orthologs. Data are shown for 1896 (Ath) and 2904 (S. rexii) “confidently annotated” representative transcripts from LSTCs. Figure S1b: box plot of percentage of transcripts corresponding to putative retained introns as estimated by parsing of Blast alignments with A. thaliana orthologs. Data are shown for 1896 (Ath) and 2904 (S. rexii) “confidently annotated” representative transcripts from LSTCs. (PDF) [file pone.0080961.s001.pdf]
